# Statistical Analysis of Mineral Concentration for the Geographic Identification of Garlic Samples from Sicily (Italy), Tunisia and Spain

**DOI:** 10.3390/foods5010020

**Published:** 2016-03-15

**Authors:** Rossella Vadalà, Antonio F. Mottese, Giuseppe D. Bua, Andrea Salvo, Domenico Mallamace, Carmelo Corsaro, Sebastiano Vasi, Salvatore V. Giofrè, Maria Alfa, Nicola Cicero, Giacomo Dugo

**Affiliations:** 1 Department of Biomedical and Dental Sciences and Morphofunctional Imaging, University of Messina, V.le F. Stagno d’Alcontres 31, Messina 98166, Italy; rvadala@unime.it (R.V.); amottese@unime.it (A.F.M.); gbua@unime.it (G.D.B.); mallamaced@unime.it (D.M.); malfa@unime.it (M.A.); 2 IPCF- CNR of Messina, Viale F. Stagno d’Alcontres 37, Messina 98158, Italy; ccorsaro@unime.it; 3 MIFT Department Physics Section, University of Messina, Viale F. S. d’Alcontres 31, Messina 98166, Italy; vasis@unime.it; 4 Department of Chemical Sciences, Biological, Pharmaceutical and Environmental, University of Messina, Via SS. Annunziata, Messina 98168, Italy; sgiofre@unime.it; 5 Science4life srl, Academic Spin-off, Università di Messina, Viale F. Stagno d’Alcontres 31, Messina 98166, Italy

**Keywords:** Nubia Red Garlic, ICP-MS, PCA, multi-element profile, geographic origin, anticarcinogenesis

## Abstract

We performed a statistical analysis of the concentration of mineral elements, by means of inductively coupled plasma mass spectrometry (ICP-MS), in different varieties of garlic from Spain, Tunisia, and Italy. Nubia Red Garlic (Sicily) is one of the most known Italian varieties that belongs to traditional Italian food products (P.A.T.) of the Ministry of Agriculture, Food, and Forestry. The obtained results suggest that the concentrations of the considered elements may serve as geographical indicators for the discrimination of the origin of the different samples. In particular, we found a relatively high content of Selenium in the garlic variety known as Nubia red garlic, and, indeed, it could be used as an anticarcinogenic agent.

## 1. Introduction

Garlic (*Allium sativum* L.) and garlic supplements are consumed in many cultures for their healthy effects on different alimentary disorders and infections, and have been since the ancient times [1]. The beneficial effects on human health are mainly provided by the organosulfur compounds [2]. Nubia red garlic (NRG), belongs to the Liliaceae family, and, in particular, to the *Allium Sativum* species. NRG is a Sicilian garlic variety and its name derives from the intense red color of the robes of its bulbils and from the denomination of the geographic area: the small district of Nubia (Paceco, Trapani) in western Sicily (Italy). In fact, the production area of NRG falls within the region of Paceco and is in a protected area: the natural reserve named “Saline di Trapani e Paceco” (SIC, ITA01007) in Northwestern Sicily, but also extends to the neighboring area of Trapani, Marsala, Buseto Palizzolo, Valderice, and Erice.

Paceco is a small rural Centre (58 km^2^) and it is considered a “clean area”, because there are not industrial centres and vehicular traffic is extremely low; the local economy is based on agriculture. The soils evolve from clay rocks with a clay content of 40% to 45% (regosols) to soils of considerable thickness and uniformity characterized by deep cracks in summer (vertisols) [3]. The climate can be defined as semi-arid with average annual rainfall of about 500 mm.

Note that the NRG has been officially added to the list of traditional Italian food products (PAT) of the Ministry of Agriculture, Food, and Forestry (M.D. 5/06/2014). NRG usually possesses a variable number (8 to 14) of tunics that are distinguished in externals (sterile tunics) with ivory-pink color that serves as protection for the cloves and internally (fertile tunics) with winey-red color from which the bulbils are formed. The bulbils are close together showing a convex dorsal surface [4].

In this paper we traced, by means of inductively coupled plasma mass spectrometry (ICP-MS), the multi-element profiles of several garlic samples of different geographical area. In the literature, there are many scientific reports of ICP-MS analysis for the determination of mineral concentration in food matrices including garlic [5,6,7,8,9,10,11]. Furthermore, nowadays there is great attention paid to the discrimination of protected foodstuffs [12,13,14]. We evaluated the statistically significant differences among the garlic samples using chemometric analyses. Furthermore, we employed Principal Component Analysis (PCA) to correlate each garlic sample to its corresponding geographic area. This approach is able to discriminate among different garlic samples depending on their mineral profiles. Finally, the concentration of some specific elements can be used to assess the quality of a specific cultivar. For example, the high level of selenium found in NRG samples suggests the ability of the corresponding garlic cultivar to block (or prevent) carcinogenesis [15,16]. Note that the presence of this element is strongly related to the compositional nature of the cultivation soil. Indeed, several Se-compounds can be found within garlic samples displaying many different beneficial health effects [17].

## 2. Materials and Methods

### 2.1. Sampling

**Figure 1:**
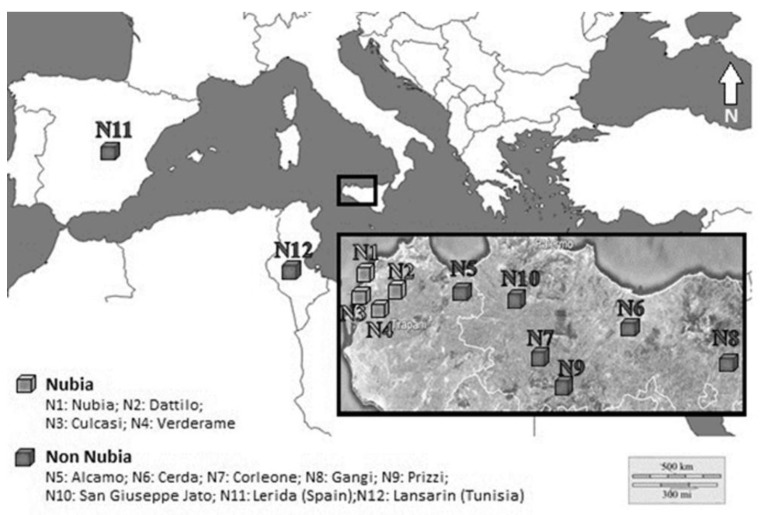
Geographic localization of Nubia and Non Nubia garlic samples.

We analyzed a total of 120 garlic samples: 40 were labeled as NRG samples and 80 as “Non Nubia” samples, following the indication reported in the introduction. Figure 1 shows their different areas of origin and Table 1 reports some general related information.

All garlic samples were harvested during the last crop season. We divided the analyzed garlic samples into two main groups. The first group named “Nubia” is composed of NRG samples (of certified origin) coming from the district of Paceco and from other neighboring areas (Dattilo, Culcasi, Verderame). The second group named “Non Nubia” is composed of garlic samples of various cultivar coming from different areas of Sicily (S. Giuseppe Jato, Corleone, Prizzi, Gangi, Alcamo, Cerda), from Lerida (Spain) and Lansarin (Tunisia). Lerida is a town located 160 km west of Barcelona whereas Lansarin is a hilly area located 30 km north of Tunis. The ICP-MS analyses were performed just on the edible part of the garlic without its internal bud.

### 2.2. Study Site

Garlic is a bulbiferous vegetable growing underground; therefore, it absorbs the minerals present in the soil. Indeed, garlic’s multi-elemental profile is strongly dependent on the composition of the soil and is mainly linked to the geological structure and soil quality of the origin site. Relative to the analyzed garlic samples, all Nubia samples come from the Paceco geographic area located in the southeast of Trapani. The soil is prevalently clayey a with high content of potassium and a low content of organic matter [18]. The other Sicilian (Non Nubia) garlic samples originate from the geographic area between Palermo (Cerda, Corleone, Gangi, Prizzi and S. Giuseppe Jato) and Trapani (Alcamo) [19]. These places are characterized by clay soils, except for Gangi, which displays a mainly arenaceous clay.

The Spanish samples come from Lerida which is characterized by sandy soils with high levels of iron [20]. The Tunisian samples come from Lansarin, which is characterized by clayey soil with high iron concentration [21].

### 2.3. Chemicals and Standard Solution

High purity gases (99.9990% argon and 99.9995% helium) were supplied by Rivoira (Rivoira S.p.A., Milan, Italy). Suprapure concentrated acids (65% *v*/*v* nitric acid and 30% *v*/*v* hydrogen peroxide, J.T. Backer, Mallinckrodt Backer, Milan, Italy) were used to digest the samples. High purity water with resistivity of 10 MΩ cm (J.T. Backer, Mallinckrodt Backer, Milan, Italy) was used throughout.

Stock standard solution (1 g/L in 2% of nitric acid) of each element under investigation (Cr, Ni, Cu, As, Se, Sb, Ba, Pb, Zn, Fe, Mg, Ca, Al, Na, K) were purchased from Fluka, Milan, Italy and (Mn and Cd) from Merck, Darmstadt, Germany. Standard solutions of Sc, Bi, In, Ge (1 g/L in 2% nitric acid) were purchased from Fluka (Milan, Italy) and were used as on-line internal standards (at the level of 1 mg/L) to correct for instrumental drift and variation due to the matrix.

To tune the instrument, an ICP-MS tuning solution containing 1 mg/L of 7Li, 59Co, 80Y and 205Tl in 2% HNO_3_ was obtained from Agilent (Santa Clara, CA, USA). Calibration standards were prepared at concentration ranges suitable for the analytes being investigated: from 0.020 to 2 mg/kg for the elements Al, As, Cd, Cr, Sb, Pb, Se; from 0.020 to 5 mg/kg for the elements Ba, Cu, Ni; from 0.5 to 50 mg/kg for the elements Ca, Fe, K, Mg, Mn, Na, Zn. The calibration curves were obtained by using five standard solutions. The internal Re standard was prepared at 0.8 μg/L. Before use, glassware was washed with 5% HNO_3_ for at the least 12 h, rinsed with ultrapure water, and then dried.

Accuracy and precision were assessed by analysing the certified standard matrices: rice flour (NIST SRM 1568a) [22]; spinach leaves (NIST SRM 1570a); cabbage powder (IAEA-359).

### 2.4. Sample Preparation

Approximately 0.5 g of fresh garlic was firstly added with 1 mL of internal Re standard at 0.8 μg/L, and then were digested with 8 mL of HNO_3_ (65% *v*/*v*) and 2 mL of H_2_O_2_ (30% *v*/*v*) in acid-prewashed PTFE vessels. The mineralization was carried out with a microwave oven at constant power (1000 W). First the temperature was increased to 180 °C in 10 min (step 1), and then it was held at 180 °C for another 10 min (step 2). After cooling down to room temperature, the digested samples were weighed. Then, 500 mg were transferred into pre-cleaned 50 mL volumetric flasks and diluted until the mark using deionized water. Finally, they were stored at 4 °C before acquisition.

### 2.5. Instrumentation

We obtained the mineralization of garlic samples by means of Ethos 1 (Milestone, Bergamo, Italy), a closed-vessel microwave digestion system equipped with sensors for temperature and pressure. The ICP-MS instrument used for the element determination is an Agilent 7500cx (Agilent Technologies, Santa Clara, CA, USA) with an MS spectrometer powered by a 27.12 MHz radiofrequency solid-state generator at 1500 W. The ICP-MS was equipped with a MicroMist glass concentric pneumatic nebulizer coupled with a cooled Scott double pass type spray chamber made of quartz. The ICP torch was a classic Fassel-type torch with wide diameter (2.5 mm) fitted with a shield torch system. Ni sampler and skimmer cones of 1.0 mm and 0.4 mm were used. An octopole collision/reaction system with helium gas was used to minimize polyatomic interferences resulting from plasma and matrix.

The equipment is provided with an off-axis ion lens, a quadrupole mass analyzer, and an electron multiplier detector. Our instrument also includes an auto sampler ASX520 (Cetac Technologies Inc., Omaha, NE, USA) and an integrated sample introduction system.

### 2.6. ICP-MS Analysis

ICP-MS is one of the most sensitive analytical techniques for fast multi-element determination of mineral elements at trace and ultra-trace concentrations in different sample matrices. The use of ICP-MS as a simultaneous multi-element detection method presents excellent selectivity and also offers high sensitivity [23]. In this paper, the instrument was tuned to achieve the best compromise between high intensities and low yields of oxidized and doubly charged ions. The operating conditions were optimized to obtain the highest signal-to-noise ratio for ^7^Li, ^59^Co, ^115^In, and ^238^U along with ^140^Ce, ^16^O/^140^Ce < 2.5% and ^140^Ce and ^16^O < 5%. In particular, we used: RF power, 1500 W; plasma gas flow rate, 15 L/min; auxiliary gas flow rate, 0.9 L/min; carrier gas flow rate 1.1 L/min; helium collision gas flow rate, 4 mL/min; spray chamber temperature, 2 °C; sample depth, 9 mm; sample introduction flow rate 1 mL/min; nebulizer pump, 0.1 rps; extract lens 1 voltage, 1.5 V. The instrument in no gas-mode for Mn, Pb, Zn, Ca, Na, K, Ba and in helium mode for Cr, Ni, As, Se, Cd, Fe, Cu, Sb, Al to remove spectral interferences has been used. Monitored isotopes were ^24^Mg, ^23^Na, ^27^Al, ^39^K, ^44^Ca, ^52^Cr, ^55^Mn, ^57^Fe, ^60^Ni, ^63^Cu, ^66^Zn, ^75^As, ^77^Se, ^111^Cd, ^121^Sb, ^137^Ba, ^206^Pb, ^207^Pb, ^208^Pb. As mentioned above, these isotopes were chosen to maximize sensitivity and to minimize interferences due to the matrix. The internal standards used were: 45Sc for Al, Na, Mg, K, Ca, Cr, Mn, Fe, Ni, Cu; ^72^Ge for Zn, As, Se; ^115^In for Cd, Sb, Ba; ^209^Bi for Pb. Integration times were 0.5 s/point for As, Cr, Cu, Ni, and Se, and 0.1 s/point for the other elements. Three points for each mass and three replicate acquisitions were taken to integrate the peaks.

The effects of ^40^Ar, ^35^Cl on ^75^As, and of ^44^Ca, ^16^O and ^43^Ca, ^16^OH on ^60^Ni were checked and the interferences were corrected by elemental interference equations. Besides, the isotopic variability in Pb was corrected by elemental interference equation. These equations, reported in various EPA methods and applied by the instrument software (ICP-MS ChemStation B.03.07, Waldbronn, Germany), contain the naturally occurring isotope ratios of elements and allow the subtraction of isobaric or polyatomic interferences.

### 2.7. Statistical Methods

All statistical calculations were made by IBM SPSS Statistics, Version 21 software package. The starting matrix was constituted by 120 rows corresponding to all the analyzed garlic samples and by 11 columns (variables) representing the concentration of the observed elements (Ba, Ca, Cu, Fe, K, Mg, Mn, Na, Se, Zn, Ni) that we found in our samples. For the preliminary statistical analysis, the data were grouped into 12 categories according to the geographic origin of samples. We calculated the average, the standard deviation, the skewness, and the kurtosis for each category. In particular, in this way we studied (i) the dispersion from the average by using the standard deviation (SD); (ii) the shape of the distribution of the collected data by means of the skewness; and (iii) a measure of the asymmetry and of the “peakedness” by means of the kurtosis. The obtained values are reported in Table 2 and confirm the goodness of the achieved data. Successively, the data in the starting matrix were subjected to Principal Component Analysis (PCA) in order to reduce data dimensionality and to cluster them depending on the total content of metals (see section Multivariate Statistical Analysis).

## 3. Results

### 3.1. Method Validation

According to international guidelines [24], the limit of detection (LOD), the limit of quantification (LOQ), the linearity, the accuracy, and the precision were determined to validate the method (Table 3). LOD and LOQ were determined as 3.3 σ/S and 10 σ/S, respectively, where “σ” is the residual standard deviation and “S” is the slope of the regression curve. Nevertheless, since a garlic standard certified matrix for trace element determination was not found in commerce, the certified references used for validation were: rice flour (NIST SRM 1568a) [22]; spinach leaves (NIST SRM 1570a); cabbage powder (IAEA-359). Precision was estimated on the relative standard deviation of measurements obtained from 10 analyses of the same certified matrices under the same operating conditions. Table 3 shows that the detection and quantification limits are adequate for the analysis; the linearity was good with R^2^ ≥ 0.9991, the accuracy and precision were also sufficiently satisfactory. Note that the accuracy can vary from 80% to 120% depending on the ratio between the measured metals concentration and that certified by the producer of the analysed matrix.

### 3.2. Multi-Element Profile of Garlic Samples

Table 2 shows the descriptive statistics on the trace elements that we detected in our garlic samples, grouped for sampling area. We did not report any values for As, Pb, Cd, Cr, Sb, or Al because their concentration was below the LOD. Nickel (Ni) was the only toxic element detected in all the studied samples. As it is well known, Ni at high doses has a toxic effect on human health. For example, the oral median lethal dose of nickel acetate was 350 mg/kg in rats and 420 mg/kg in mice [25,26]. However, the Nickel concentration found in all garlic samples are very low and cannot cause any injury to human health.

Indeed, Nickel concentration could serve as a biomarker for geographical origin. In fact, from the inspection of Table 2, the garlic samples coming from Tunisia and Spain possess the highest Ni level. Moreover, particularly significant is the comparison between Selenium (Se) levels in the analyzed samples. The concentration of this element is higher in NRG samples compared to Non Nubia samples.

### 3.3. Multivariate Statistical Analysis

**Figure 2:**
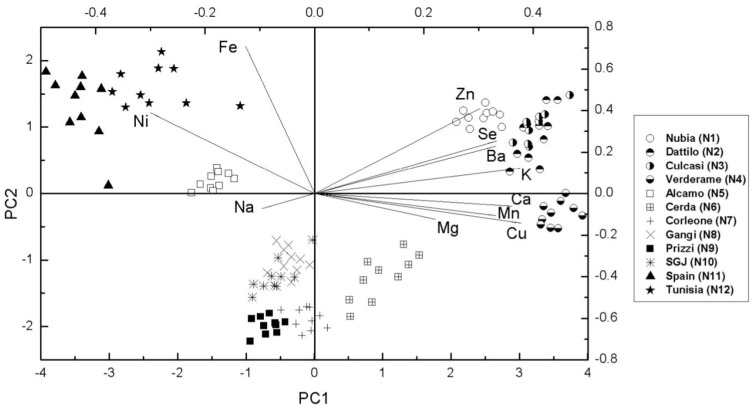
Score (**left** and **bottom** axes) and loadings (**right** and **top** axes) plot showing the results of the PCA performed on all the analyzed garlic samples.

As mentioned before, we performed a multivariate statistical analysis in terms of the Principal Components Analysis (PCA). It is an “unsupervised” statistical method that can reduce data dimensionality and allows data clustering with respect to the considered variables. Data were mean-centered and the singular value decomposition (SVD) algorithm was applied to perform a PCA with cross validation. The PCA technique allowed the reduction of the data dimensionality from 11 to 2 with a variance percentage higher than 70%. Figure 2 reports the resulting score plot, together with the corresponding loadings (top and right axes), of the first two principal components (PC1 *vs.* PC2). The score plot illustrates how this kind of analysis is able to discriminate the different garlic species, being able to cluster and group the sample depending on the relative content of metals. The corresponding loadings plot (top and right axes of Figure 2) is used to identify the bases of the clustering. In particular, samples from Nubia (N1) and Dattilo (N2) can be mainly discriminated because they show the highest levels of Zn and Se. In fact, as shown in Table 2, for these samples the average concentration of Zn is 17.63 and 18.8**4** mg/kg, and the average concentration of Se is 0.244 and 0.221 mg/kg, respectively. Samples from Culcasi (N3) show the highest content of Ba and Cu (0.860 and 3.261 mg/kg, respectively) whereas those from Verderame (N4) show the highest content of Ca and Mn: 1823 and 13.336 mg/kg, respectively. Finally, note that the samples from Spain and Tunisia show the highest content of Fe (25.10 and 29.97 mg/kg, respectively) and Ni (0.669 and 0.651 mg/kg, respectively).

## 4. Discussion

On average, garlic contains approximately 65% water, 28% carbohydrates (fructans), 2.3% organosulfur compounds, 2% proteins (alliinase), 1.2% free amino acids (e.g., arginine) and 1.5% fibers. Allicin is the main active substance that is responsible for the typical pungent smell and for garlic’s therapeutic properties [27,28,29]. When fresh garlic is chopped or crushed, alliin (a sulfoxide that is a natural constituent of fresh garlic) transforms into allicin by the action of an enzyme called alliinase. However, allicin is an unstable compound and easily oxidable into a series of other sulfur-containing compounds such as diallyl disulfide. Furthermore, for condensation reactions, allicin can be converted into ajoenes [30], into vinyldithiins [31], and into thiosulfinates that also have a good curative action [32]. Besides allicin, garlic contains different compounds able to produce many beneficial effects for health. For this reason, garlic is used not only as flavoring but also for healing in many cultures [33].

The overall results of our analysis show that the application of Principal Component Analysis to the measured minerals content allows for sample discrimination. Samples of NRG show an average content of Se, K, Zn, and Ca that is higher than Non Nubia samples and an extremely low Ni concentration, especially if compared with samples from Spain and Tunisia. Note that just the Selenium levels are one order of magnitude higher in NRG samples. Since many healthy beneficial effects of garlic are related to the presence of different Seleno-compounds [17], this suggests the use of garlic and in particular of that coming from the Paceco district for preventing or blocking carcinogenesis. In fact, the association between reduced risk in intestinal cancer and high intake of garlic was investigated by the European prospective investigation into cancer and nutrition (EPIC). Several epidemiological studies suggest that the consumption of garlic and related allium foods reduces the risk of certain cancer types, including those of the gastrointestinal tract [34,35]. The potential benefits of garlic as an anticancer agent is indeed mainly due to its ability to accumulate Selenium, which is a cancer fighting mineral.

## 5. Conclusions

In this work, we determined the multi-elemental profiles of different garlic samples by ICP-MS analysis in order to individuate through a multivariate statistical analysis which elements may be considered as geographic indicators for the discrimination of origin. We analyzed four garlic varieties coming from the district of Paceco (TP, Sicily, Italy), known as Nubia Red Garlic (NRG) and certified as traditional Italian food products (P.A.T.) by the Ministry of Agriculture, Food, and Forestry [36], and compared with six other varieties coming from different places within Sicily and with two varieties coming from Spain and Tunisia. Garlic is widely consumed in different parts of the world and many studies report its healthy protective effect against many different diseases, independent of the specific cultivar and from the particular geographic origin of the garlic. The healthy effects of garlic depend on the level of some mineral elements, such as Se, K, Zn, and Ca. In particular, the high content of Selenium found in NRG samples identifies this element, not only as an indicator for the discrimination of geographic origin of this cultivar, but it is also useful to demonstrate that Nubia Red Garlic shows important health qualities and could be used as an anticarcinogenic agent.

## Figures and Tables

**Table 1:** General information about the studied garlic samples.

	Sample Source	Latitude North	Longitude East	NS ^a^	Bulb Das ^b^ (mm)	Bulb Aw ^c^ (g)	Bulbils An ^d^	Bulbils Aw ^c^ (g)
NRG SAMPLES								
N1	Nubia	37°58′43.58′′	12°30′42.27′′	10	50	45.21	13	3.12
N2	Dattilo	37°58′13.68′′	12°38′20.62′′	10	48	44.92	13	3.33
N3	Culcasi	37°58′36.98′′	12°29′57.53′′	10	49	44.72	13	3.24
N4	Verderame	37°58′10.96′′	12°32′36.88′′	10	52	45.63	13	2.96
	NON NUBIA SAMPLES							
N5	Alcamo	37°58′39.26′′	12°58′33.76′′	10	48	42.86	13	3.19
N6	Cerda	37°54′12.80′′	13°48′53.48′′	10	45	43.56	13	3.25
N7	Corleone	37°48′42.31′′	13°17′39.11′′	10	43	39.2	11	2.86
N8	Gangi	37°47′34.42′′	14°11′59.66′′	10	45	44.02	12	3.56
N9	Prizzi	37°42′53.15′′	13°25′49.59′′	10	47	43.23	12	3.51
N10	San G. Jato	37°58′07.17′′	13°10′41.08′′	10	46	41.71	12	3.37
N11	Lerida (Spain)	41°35′08.58′′	0°42′18.85′′	10	55	51.03	10	4.97
N12	Lansarin (Tunisia)	37°03′46.60′′	10°06′33.33′′	10	47	42.73	12	3.26

^a^ NS, Number of Samples; ^b^ das, diameter average size; ^c^ aw, average weight; ^d^ an, average number.

**Table 2:** General information about the studied garlic samples.

		Sample Source		Elements Concentration (mg/kg)										
Nubia Rec Garlic Samples	N1	Nubia		Ba	Ca	Cu	Fe	K	Mg	Mn	Na	Se	Zn	Ni
			Mean	0.518	1800	2.531	23.10	5920	269.7	10.39	98.56	0.244	17.63	0.048
			Std. Dev.	0.096	110.8	0.211	1.156	36.80	5.814	0.702	4.685	0.430	1.767	0.007
			Skewness	−0.150	−0.378	0.308	−0.037	0.951	−0.106	0.431	−0.084	0.059	0.107	0.089
			Kurtosis	0.815	−0.501	−1.331	0.083	0.109	−0.715	0.551	−1.25	−1.489	−1.06	−0.849
			Range	0.328–0.636	1585–1976	2.246–2.860	21.07–25.00	5877–5988	259.5–379.6	9.40–11.87	91.63–105.8	0.187–0.313	14.67–20.38	0.037–0.061
	N2	Dattilo	Mean	0.546	1723	2.843	20.76	7154	254.4	13.08	110.9	0.221	18.84	0.047
			Std. Dev.	0.116	92.57	0.095	0.904	178.57	8.577	0.405	7.446	0.023	2.838	0.006
			Skewness	0.576	−0.118	−0.421	1.099	−0.190	−1.105	0.511	−0.102	0.873	0.061	0.899
			Kurtosis	0.021	−0.413	0.848	1.714	−0.346	1.138	0.357	−0.893	0.844	0.688	0.849
			Range	0.398–0.771	1575–1875	2.652–2.996	19.76–22.76	6849–7441	235.9–263.6	12.44–13.86	98.61–119.2	0.194–0.271	14.90–23.69	0.038–0.061
	N3	Culcasi	Mean	0.860	1740	3.261	24.10	6253	264.02	13.250	114.43	0.152	16.56	0.042
			Std. Dev.	0.107	138.67	0.191	0.724	277.79	4.72	0.461	10.065	0.018	2.014	0.008
			Skewness	−0.651	−0.446	−0.218	−0.142	0.669	0.639	0.053	−0.332	−2.065	−0.561	0.263
			Kurtosis	−1.414	−1.486	−1.569	−1.076	−1.173	−1.415	−1.267	−0.364	5.229	0.0077	−1.398
			Range	0.685–0.976	1523–1911	2.998–3.498	23.08–25.16	5978–6742	259.16–271.42	12.55–13.81	99.16–131.01	0.104–0.171	12.91–19.69	0.031–0.055
	N4	Verderame	Mean	0.687	1823	3.110	15.671	7041	282.95	13.336	97.06	0.165	15.31	0.047
			Std. Dev.	0.132	119.86	0.134	1.442	141.28	10.295	0.794	3.072	0.41	1.437	0.008
			Skewness	−0.535	0.075	0.402	−1.444	−0.620	0.468	−1.517	−0.721	0.458	−0.591	−1.055
			Kurtosis	−0.983	−1.239	−0.834	2.287	1.664	−1.099	0.935	0.205	0.604	2.281	0.631
			Range	0.473–0.850	1653–1989	2.915–3.332	12.34–17.00	6740–7249	270.42–300.01	11.77–13.98	90.95–101.30	0.110–0.248	12.26–17.84	0.029–0.057
Non Nubia Samples	N5	Alcamo	Mean	0.211	1274	0.946	24.63	4349	245.56	7.529	94.13	0.196	9.249	0.122
			Std. Dev.	0.023	235.94	0.062	1.423	307.04	25.529	0.356	11.96	0.003	1.495	0.015
			Skewness	−0.754	−0.541	−0.491	−0.462	0.557	1.554	−0.777	−1.874	−0.221	1.047	0.765
			Kurtosis	−1.082	−3.232	−2.295	−2.462	−2.457	2.322	−2.242	3.74	−1.317	1.998	0.182
			Range	0.178−0.236	1010–1478	0.867–1.012	22.81–25.96	4080–4746	225.79–287.98	7.04–7.89	73.45–103.25	0.015–0.024	7.570–11.62	0.105–0.146
	N6	Cerda	Mean	0.332	1592	2.811	17.89	4663	277.53	12.204	100.698	0.045	8.993	0.468
			Std. Dev.	0.11	95.30	0.127	0.183	138.701	10.368	1.18	1.493	0.015	2.653	0.337
			Skewness	−0.794	−0.446	−1.533	0.605	0.044	−0.621	−0.806	0.487	−0.235	0.053	−0.552
			Kurtosis	−1.833	−0.263	2.388	−1.601	−1.927	−1.011	−1.703	−0.463	−0.922	−2.5	−3.216
			Range	0.178–0.426	1456–1701	2.600–2.917	17.72–18.14	4522–4840	264.37–290.84	10.54–13.25	98.98–102.81	0.025–0.064	5.923–12.00	0.066–0.117
	N7	Corleone	Mean	0.220	1515	2.201	13.74	3442	248.57	11.045	119.20	0.045	8.113	0.057
			Std. Dev.	0.042	176.35	0.274	0.192	573.01	29.681	0.606	3.529	0.014	0.686	0.022
			Skewness	0.188	−1.303	0.307	0.277	0.461	−1.938	0.424	−0.531	−0.192	1.188	−0.678
			Kurtosis	−1.847	0.562	−1.458	−0.137	−3.082	3.849	−1.581	−0.185	1.461	1.370	1.054
			Range	0.175–0.275	1237–1636	1.875–2.560	13.50–14.01	2875–4087	197.00–269.31	10.41–11.86	114.11–123.21	0.025–0.065	7.500–9.200	0.024–0.084
	N8	Gangi	Mean	0.222	967.0	2.114	17.25	4304	294.78	8.815	84.075	0.028	9.515	0.112
			Std. Dev.	0.083	56.51	0.179	0.228	353.74	7.382	0.241	30.256	0.008	1.282	0.031
			Skewness	0.588	0.361	0.037	−0.922	0.824	0.034	−1.536	2.160	1.886	−0.770	0.005
			Kurtosis	−3.183	−0.600	−2.545	1,154	−0.432	0.006	2.319	4.728	3.859	−2.266	−0.529
			Range	0.150–0.320	899.0–1045	1.913–2.322	16.89–17.50	3981–4827	284.98–304.65	8.41–9.02	65.71–137.82	0.021–0.043	8.160–11.08	0.070–0.153
	N9	Prizzi	Mean	0.190	1016	1.959	13.70	4294	275.36	10.197	135.75	0.05	6.628	0.073
			Std. Dev.	0.032	63.90	0.098	0.371	244.41	16.544	0.476	10.43	0.0190	0.679	0.015
			Skewness	−0.353	0.473	−1.919	−1.429	0.071	−1.709	0.825	−0.265	−1.185	1.651	0.363
			Kurtosis	−2.309	−2.158	4.050	2.113	−2.73	3.161	−1.096	−1.517	1.658	3.099	−2.662
			Range	0.150–0.225	947.0–1098	1.787–2.040	13.08–14.00	4023–4566	247.25–289.20	9.77–10.88	121.86–147.21	0.019–0.070	6.016–7.777	0.056–0.092
	N10	San G. Jato	Mean	0.211	1447	1.806	14.23	3029.4	184.20	10.489	113.90	0.067	11.69	0.061
			Std. Dev.	0.028	76.10	0.040	0.526	59.79	3.99	0.531	5.56	0.016	4.109	0.012
			Skewness	0.316	0.900	1.740	0.885	1.529	0.742	−0.003	0.829	−0.334	2.066	−1.519
			Kurtosis	−2.156	0.918	3.256	−0.734	2.561	−0.573	−2.31	0.992	−2.132	4.335	2.445
			Range	0.117–0.246	1365–1564	1.776–1.875	13.72–14.99	2980–3129	180.11–190.01	9.87–11.12	107.62–122.40	0.046–0.085	9.200–18.92	0.040–0.071
	N11	Spain	Mean	0.216	804.6	0.960	25.10	2898	164.08	6.347	132.46	0.03	10.23	0.669
			Std. Dev.	0.086	107.16	0.144	0.260	185.30	10.13	0.399	34.548	0.01	2.446	0.262
			Skewness	1.275	0.419	0.802	0.640	−0.845	−0.379	0.489	0.099	0.343	−0.376	−1.284
			Kurtosis	2.721	−1.375	0.317	−0.393	−0.306	0.651	−1.549	−2.34	−0.457	−0.245	1.567
			Range	0.098–0.415	675.0–982.0	0.764–1.245	24.78–25.57	2537–3088	145.96–181.52	5.92–6.98	96.52–176.21	0.015–0.051	6.160–13.94	0.092–0.910
	N12	Tunisia	Mean	0.187	698.2	1.137	29.97	3268	270.61	10.173	97.55	0.017	9.350	0.651
			Std. Dev.	0.089	64.418	0.158	0.326	201.8	10.976	0.577	5.555	0.005	2.785	0.299
			Skewness	0.112	0.422	0.381	2.285	−0.074	0.385	−0.549	−1.089	1.05	0.637	−1.373
			Kurtosis	−1.978	−1.560	−1.031	5.813	−0.993	0.083	0.171	1.743	0.188	−0.825	0.558
			Range	0.105–0.300	618.0–798.0	0.957–1.412	29.69–30.81	2968–3555	251.54–288.28	9.02–10.84	85.27–104.25	0.012–0.027	5.94–13.74	0.102–0.910

**Table 3:** Analytical performance of the method.

Element	Isotope	LOD ^a^ (mg/kg)	LOQ ^b^ (mg/kg)	Calibration Range (mg/kg)	R ^2 c^	Precision (SDR%, *n* = 10) ^d^	Accuracy ^e^ (%)
Al	27	0.015	0.052	0.020–2	0.9998	2.9	85.63
As	75	0.010	0.035	0.020–2	0.9996	2.6	89.78
Ba	137	0.015	0.052	0.020–5	0.9999	3.7	90.71
Ca	44	0.017	0.059	0.5–50	0.9993	2.9	101.54
Cd	111	0.018	0.063	0.020–2	0.9999	2.7	94.53
Cr	52	0.011	0.038	0.020–2	0.9998	2.4	91.78
Cu	63	0.018	0.063	0.020–5	0.9999	2.1	86.56
Fe	57	0.017	0.059	0.5–50	0.9999	2.2	96.54
K	39	0.016	0.056	0.5–50	0.9991	3.6	102.31
Mg	24	0.015	0.052	0.5–50	0.9993	3.3	101.44
Mn	55	0.014	0.049	0.5–50	0.9999	2.9	102.9
Na	23	0.016	0.056	0.5–50	0.9997	2.6	86.76
Ni	60	0.010	0.035	0.020–5	0.9999	3.2	83.32
Sb	121	0.012	0.042	0.020–2	0.9999	2.8	93.68
Pb	208	0.011	0.038	0.020–2	0.9999	2.1	94.37
Se	77	0.010	0.035	0.020–2	0.9995	2.5	85.89
Zn	66	0.016	0.056	0.5–50	0.9999	3.1	97.32

^a^ LOD, limit of detection (3.3 σ/S); ^b^ LOQ, limit of quantification (10 σ/S); ^c^ R^2^, least square regression coefficient; ^d^ SDR, Standard Deviation Ratio; ^e^ Average of 10 replicates.

## Abbreviations

The following abbreviations are used in this manuscript:

NRG	Nubia Red Garlic
PAT	Product agriculture Traditional
ICP-MS	Inductively Coupled Plasma Mass Spectrometry
PCA	Principal Components Analysis
LOD	Limit of Detection
LOQ	Limit of Quantification
SVD	Singular Value Decomposition
PC	Principal Component
